# Identifying positions and roles of travel agencies based on relationship redundancy in a package tour network

**DOI:** 10.1016/j.heliyon.2020.e03227

**Published:** 2020-01-20

**Authors:** Yu-hsin Chang

**Affiliations:** Department and Graduate Institute of Marketing and Logistics Management, Chaoyang University of Technology, Taiwan

**Keywords:** Package tour (PAK), Social network analysis, Relationship redundancy, Active and complementary relationship, Position and role, Tourism, Business

## Abstract

The travel agencies have a cooperative-competitive relationship with each other. In line with the background, the agencies need to know clearly their positions and roles in the industrial network. First, an affiliate network matrix is established based on the relationship between travel agents that participate in the package tour (PAK). Then, the two-mode network analysis and relationship redundancy are adopted to construct two new indicators, namely, the active relationship and complementary relationship, to assess the agencies’ positions and roles in the tourism network. The active relationship (AR) is constructed to identify the activeness of an agency in the travel industry while the complementary relationship (CR) is constructed to identify the need of a travel agency for complementary resources. A two-dimensional coordinate chart is formed to analyze the positions and roles of the travel agencies. The travel providers in this study are classified into followers, speculators, dependents and leaders.

## 1 Introduction

With the changes in leisure time, social values, and the economic market, the deepening globalization and advances in high technology increased the income of the public worldwide. The rise in income stimulates their consuming power and boosts their need for tourism. Traveling abroad is extremely prevalent in Taiwan, an island. According to statistical data from the Taiwan Tourism Bureau, the number of tourists traveling abroad has increased significantly from 8,208,125 in 2005 to 14,588,923 in 2016, which accounted for more than 60% of the Taiwanese population of around 23 million. Consumers’ demands for tourism are constantly changing. Therefore, travel providers must develop diverse tourism products that meet market demands and provide better quality services that customers are willing to pay.

Usually, tourism products are not protected by patent rights. In the tourism industry, tourism products are often imitated among the peer companies. As the market changes, travel agencies need to transform their operation (Ghorbani et al., 2019; Lerner and Haber, 2001). Travel agencies that mean to maintain their competitive advantages and survive the fierce competition must have its own unique, inimitable and valuable resources (Denicolai et al., 2010; Peteraf, 1993; Wartini-Twardowska and Twardowski, 2019; Wernerfelt, 1984). For many travel providers, they want to retain their unique resources and imitate others at the same time so that the complementary resources between upstream and downstream players can be integrated (Burkhard et al., 2012; Teece, 1986; Teece, 2010). Moreover, parallel competitors share the market with each other and pool the clients to organize tour groups. In this way, travel agencies form a special cooperative and competitive relationship between each other. In response to their interdependency on the resources among industry players, Japanese travel agencies jointly promote the package tour, hereinafter referred to as PAK. They become alliances through PAK to share complementary resources by combining the resource advantages of the partners to achieve business goals (Das and Teng, 2003; Haberli Junior, Oliveira, Yanaze and Spers, 2019). With the increasingly frequent cooperation through PAK in the travel industry, the travel providers are looking for partners in the face of the highly competitive tourism market. Under such a delicate cooperative and competitive relationship, the travel providers’ understanding of its positions and roles in the industrial network helps them to adjust their competitive strategy.

With the development of the concept of Social Network Analysis (SNA) and technologies, the SNA renders the organization structured, describes the evolution of the relational structure between organizations, and presents their relationships in a network diagram (White et al., 2004). The two-mode affiliate network analysis contains a group of actors and a set of affiliate events. This study establishes an affiliate matrix between the travel providers and the PAK, with the travel agency as the actor and PAK as the event. In this way, the affiliate matrix and its transposed matrix are calculated to establish new indicators with relationship redundancy, including self-related redundancy and other-related redundancy. The indicators are constructed to identify the position and role of a travel agency and its opponents in the network. This study expects to achieve the following purposes.

1. The degree of centrality of the Social Network Analysis is used to propose two new indicators to assess competitive status.
2. Travel providers are to understand their position and role in the tourism industry.
3. The study results can serve as references for the decision-making of the travel providers who mean to formulate a suitable strategy to improve their positions in the industry.

## 2 Concept establishment

The travel industry is a service industry in which individuals or corporations sell travel related products to the general public, including accommodation, catering, transportation, and sightseeing (Wartini-Twardowska and Twardowski, 2019). The travel industry not only shares the characteristics of the service industry, but also serves as an intermediary in the tourism industry. Therefore, the tourism industry is untouchable, changeable, non-storable, indivisible, competitive, professional, and integrated. Moreover, its related businesses are of rigidity and its demands are of seasonality and instability.

### 2.1 Package tour (PAK)

As the market changes, the highly competitive travel industry must be constantly transformed (Burkhard et al., 2012). Japan's travel agencies jointly promote Package Tour (PAK). At first, the airlines formed a PAK team with the travel agencies so that they could develop new routes regularly or increase the overall passenger-loading factor during the off-season. In other countries, different seasons present significant differences in the tourism demand, i.e. on-season and off-season. In Japan, the PAK was mainly expected to overcome the non-storability and seasonality in tourist demands of the service industry.

The market competition was fierce and the needed market space was also expanding. In that case, it was necessary to integrate resources, make full use of resources and diversify the development channels. That is why the PAK was established. Through the PAK, travel providers and airlines can cooperate to promote customized tourism by combining the travel agencies and local tourism resources. In this way, an organization with joint sales, centralized operations and sharing benefits is formed. The operating modes of the PAK are as follows. (1) Unified operation: a travel agency is selected as an operation center among PAK members. Usually those who are rich in operation experience are selected to operate PAK, or the operation work is assigned or rotated among the members. (2) Individual operation: PAK members are independent from the travel agencies. The finance is also independent. This combination provides a relatively weak power to the members. Its profit distribution can be generally divided into performance distribution, profit sharing and financial independence. However, benefit distribution is mostly carried out based on their actual performance, which is based on the actual number of heads that each travel agency allocates for the PAK.

Due to the business model of PAK, most of the PAK members are small and medium-sized enterprises. Due to limited resources and market restrictions (BarNir and Smith, 2002), Taiwan's travel industry integrates and makes full use of resources and diversifies the development channels to increase the market demand. Moreover, travel providers, or the travel agencies and airlines can cooperate to promote customized tourism service by combining the travel agencies and local tourism resources. As well, an organization with joint sales, centralized operations and sharing benefits is formed for different types of tourism or groups.

### 2.2 Social network analysis

Social Network includes a set of actors and their ties or relationships (Dev et al., 1996; White et al., 2004). Relationships may include relatives, roles within an organization, social relationships of partners, and other types of relationships. The collection of these relationships forms a social structure. There are one-mode and two-mode data structures in the network analysis. The one-mode network analysis is to measure the relationship between groups of actors of the same nature while the two-mode data can measure two types of relationships. The first category is the relationship between two actors of the different nature; a set of actors sends links to another set. The set of actors who usually sends links is called “senders” while the other set who is responsible for receiving the links is “receivers” (Wasserman and Faust, 1994). The second category is the affiliate relationship between actors and events (BarNir and Smith, 2002), such as the relationship between the company's board of directors and members (Ghorbani et al., 2019), the relevant research on the relationship between user and Facebook (Ozimek et al., 2017), firms engaging in alliances (BarNir and Smith, 2002), and community for smoking cessation (Cobb et al., 2010).

**Figure 1:**
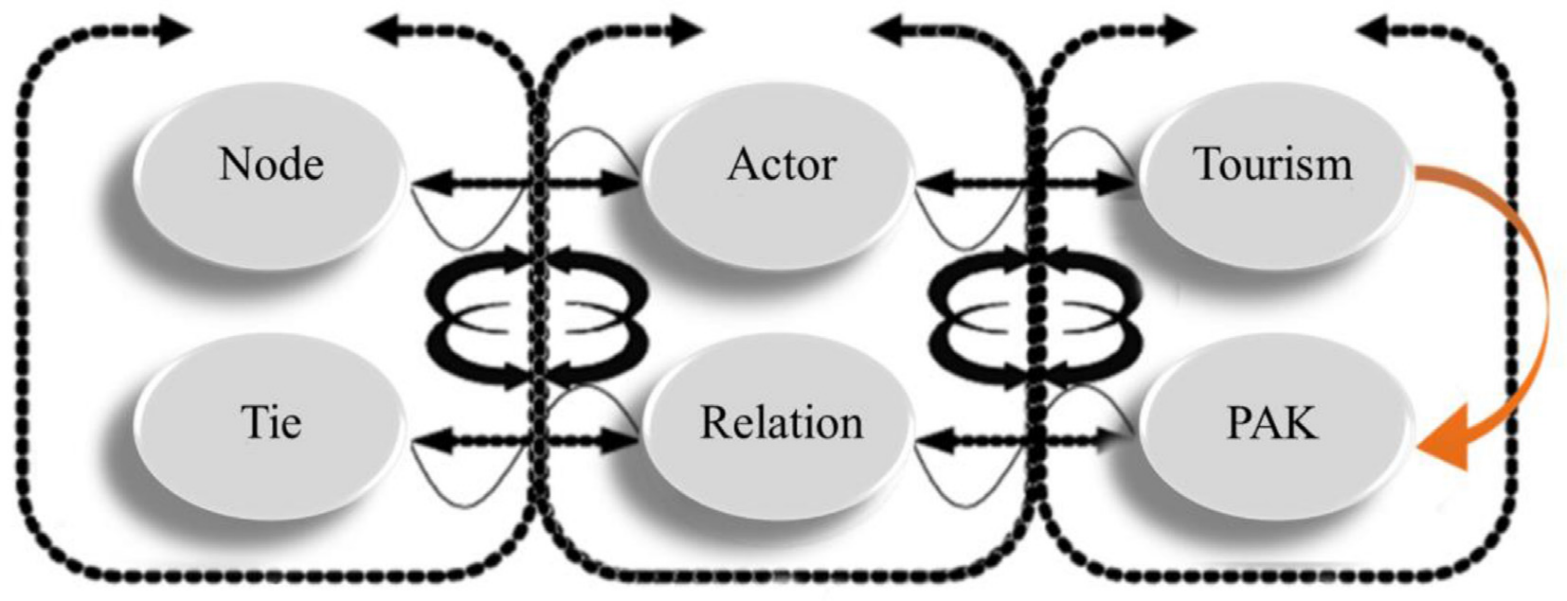
Comparison of the affiliation of the travel industry in social networks.

In this study, the affiliate relationship between actors and events is adapted as the data structure, a set of travel providers as actors and PAKs as affiliate events, as shown in Figure 1. An affiliate matrix is established with the affiliate relationship.

### 2.3 Relationship redundancy and resource complementarities

A two-mode affiliate matrix with actors and events is established, after which the affiliate matrix is transposed to an event-actor matrix. After the event-actor matrix is multiplied to the affiliate matrix, a one-mode actor-actor adjacency matrix is obtained, which is a symmetric matrix. The diagonal of this matrix is the self-related redundancy counts generated by the actors through the event. The sum of the row or column matrix that subtracts the diagonal represents the other-related redundancy counts generated by the diagonal actors and other actors through the event. These are called relationship redundancy.

The relationship between enterprises is no more than two kinds, i.e. cooperating and competing to obtain resources and determine what resources should be acquired (Abrahamsen et al., 2012). Product innovators are not necessarily the winners in the market and followers with complementary resources may be more competitive (D. J. Teece, 1986).

Complementary resources refer to the resources provided by a partner and considered unique by other partners (Dymsza, 1988). The competitive advantage of a company derives from mutually complementary resources that the partners have, which facilitates the creation of more new energies (Boonstra et al., 2015). The exchange of complementary resources can spread risks, increase market influence, remove barriers to entry and increase economic sizes, Research and Development activities, and production activities, etc (Boonstra et al., 2015; Lozada et al., 2019). Seen from the perspective of resource dependence, complementary resources are the ones needed for survival and reduce environmental uncertainties (Haberli Junior et al., 2019). Therefore, enterprises will evaluate the complementary resources that they need and conduct strategic alliances to ensure the availability of resources for survival and to reduce environmental uncertainties. Only in this way can they synergize and have a lasting competitive advantage.

This study makes a travel agency-PAK matrix with the travel agency as an actor and the PAK as an event. After the matrix is transposed and then multiplied to the original matrix, another matrix is obtained, revealing the relationship between the travel agencies. The diagonal of the matrix is the redundancy relationship between the travel providers and it also represents how many times it has participated in the PAK in total. The row matrix is the redundancy relationship between a travel provider and others through the PAK, representing the total count the diagonal companies and others jointly take part in the PAK. The matrix enables a travel provider to understand his own capabilities and his competitors’ as well as his need for complementary resources.

### 2.4 Positions and roles

The concepts of positions and roles are used to analyze the structural similarities and patterns of relationships among actors (Wasserman and Faust, 1994). Generally, the analysis is conducted first to establish the social positions of the actors and then further explore their social roles. Social position refers to the ensemble of actors and the social role is the behavior of an occupant of a social position to others (DiMaggio, 1992; Wasserman and Faust, 1994). The role is usually affiliated to a given social position, and social expectations regulate the behavior of the occupants of a given social position (Abrahamsen et al., 2012; Ozimek et al., 2017). The actors are classified into several positions in a network structure through position analysis while the interrelationship between different positions is explored through role analysis and each role is given an appropriate name.

This study will explore the PAK set by travel providers, with which tourism products and resources are jointly developed and benefits are shared. Further, the analysis of positions and roles is to be conducted with the PAK as relationships and travel providers as actors, active relationship and other relationship-related redundancy to complementary relationship as indicators for assessment criteria.

## 3 Research methods

**Figure 2:**
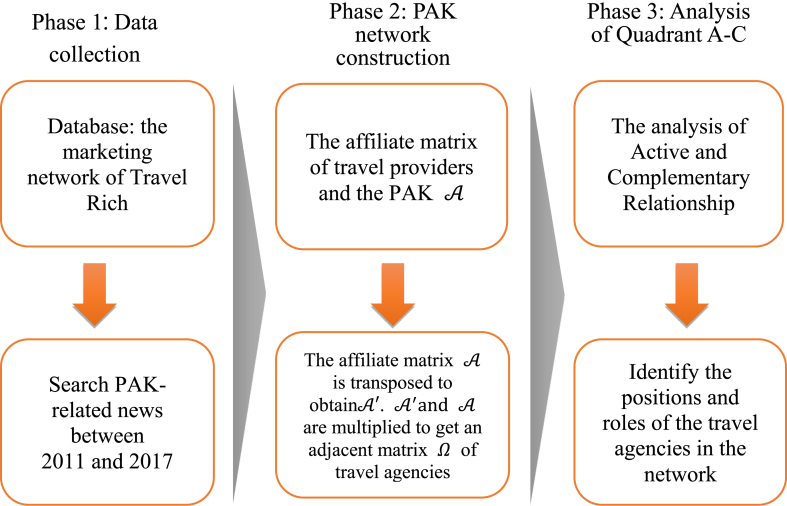
Research process architecture.

The research consists of three phases. On Phase 1, data are collected. After the collation, a PAK data set for the travel industry is formed. On Phase 2, a network of the relationship between the travel providers and the PAK is constructed. The network analysis is performed through NetMiner. After that, the centrality of the network is adopted to understand the structural nature of the tourism industry network and select important companies as analytical samples. On Phase 3, the indicators of AR and CR are constructed to carry out matrix analysis, through which the characteristics of the group are identified to understand the roles and the impact between the travel providers, as shown in Figure 2.

Phase 1 Data Collection

Research objectives and research focuses are set first. Later, the search is conducted through “Travel Rich Marketing Information Network” and “PAK” as the keywords to conduct searches and to compile data. In this way, a PAK relationship data set for the tourism industry is formed.

Step 1 Database–Travel Rich Marketing Information Website

“Travel Rich Marketing Information Network” is the most trusted representative travel provider in Taiwan's travel industry, covering the media agency, integrated marketing, tourism marketing trends and tourism marketing information network for consumer and the industry. It is a travel platform of both depth and breadth, intercepting effective travel information in most accurate manner. In addition, the professionalism of the travel industry is enhanced through specialists analyzing marketing experience.

Step 2 PAK-related news between 2011 and 2017 searched with “PAK” as the keyword

The “PAK” is the most important link between the travel providers who take part in the package tour. Therefore, PAK is set as the keyword. The date of data retrieval is mainly based on the news release date. The data retrieval period is between January 1 of 2011 and March 31 of 2017. In total, 252 data entries are retrieved on March 31 of 2017. After the data are collected, repeated PAK data and non-travel providers (airlines, foreign travel agencies, and foreign tourism bureaus) are excluded. As a result, 188 out of 252 entries remained, which involve 332 travel providers. The data set of the 188 entries $A_{332\times 188}$ is obtained.

Phase 2 Constructing a PAK Network.

Social network analysis is used to understand the subtle relationship between large travel providers and small and medium ones in the industry. Accordingly, an affiliate network matrix of travel providers and PAK events A is constructed. Then, its adjacent matrix is transposed. After that, the transposed matrix and the original matrix A are multiplied. In this way, a symmetric matrix between travel providers is obtained. Lastly, the symmetric matrix is used to calculate the number of its centrality through NetMiner.

Step 1 Establish an affiliate matrix of the travel providers and the PAK.

**Figure 3:**
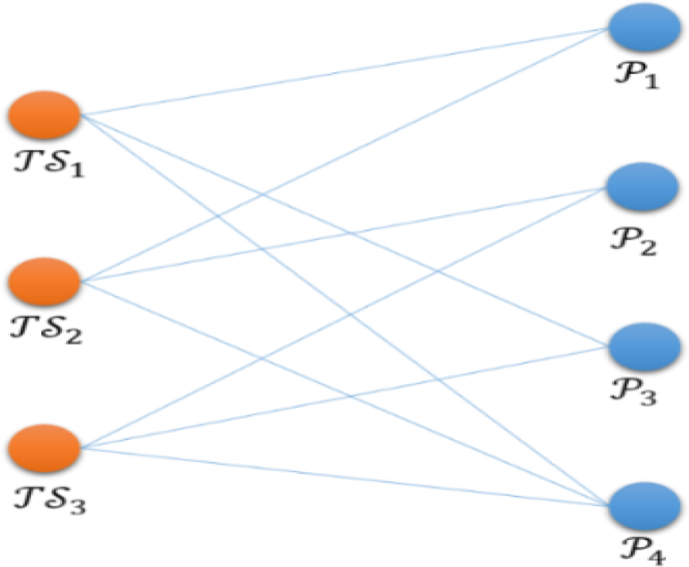
Schematic diagram of an affiliate network.

The set of the affiliate network is defined as A={TS,P}, where TS={TravelAgency} and P={PAK}. The symbols for the PAK relationship between the travel providers are defined as $P_{1}=\left\{TS_{1},TS_{2}\right\}$$P_{2}=\left\{TS_{2},TS_{3}\right\}$$P_{3}=\left\{TS_{1},TS_{3}\right\}$, and $P_{4}=\left\{TS_{1},TS_{2},TS_{3}\right\}$. The symbols mean that the $TS_{1}$, $TS_{2}$, and $TS_{3}$ agencies are linked through PAK. The corresponding schematic matrix is shown in Table 1 while the schematic diagram is shown in Figure 3.

**Table 1:** Schematic matrix of an affiliate network.

	$P_{1}$	$P_{2}$	$P_{3}$	$P_{4}$
$TS_{1}$	1	0	1	1
$TS_{2}$	1	1	0	1
$TS_{3}$	0	1	1	1

According to the retrieved PAK data set, an affiliate matrix$A_{332\times 188}$ is constructed, as shown in Eq. (1).

(1) $$A_{332\times 188}=\left\{\alpha_{ij\;}\text{|}\begin{array}{c} No.TS_{i}\text{​enterprise ​takes ​part ​in ​No ​}P_{j}\text{ ​PAK ​}\\ i＝1,2,\ldots ,332,\;j＝1,2\ldots ,188 \end{array}\right\}$$

Step 2 The adjacent matrix of travel providers Ω

After the original matrix$A_{332\times 188}\;$is transposed, the transposed matrix $A_{188\times 332}^{\text{'}}$ is obtained. After the transposed matrix$\;A^{'}$is multiplied to the original matrix A, Ω=A'×A is obtained, and Ω is the adjacent matrix of the travel providers, as shown in Eq. (2).

(2) $${\text{Ω}}_{332\times 332}=A\text{'}\times A=\left\{r_{mn\;}\text{|}\begin{array}{c} TS_{m}\text{ ​and ​}TS_{n}\text{partipate ​in ​the ​PAK ​jointly}\\ m＝1,2,\ldots ,332,\;n＝1,2\ldots ,332 \end{array}\right\}$$

Phase 3 Analysis of AR and CR Based on the Quadrants

This study constructs two indicators based on the position analysis, i.e. the active relationship derived from self-related redundancy and the complementary relationship derived from other-related redundancy. These two indicators are further used to draw the Active-Complementary graph, which will be used to analyze the position and role of the companies in the four quadrants.

Step 1 Analyze the corporate activeness and complementarities
Active relationship

The value of the diagonal of the adjacent matrix represents self-related redundancy, which shows how many times travel providers participate in the PAK. That is why it is defined as the activeness of the corporate relationship. The higher the value is, the more frequent a travel provider participates in the PAK. If a provider has a value of above average, it means that the provider is relatively active throughout the industry. Such a travel provider is very likely to have core resources or diversified resources of channels in the market. Due to its competitive position in the tourism industry, it is more likely to control the entire target market and has a variety of competitive strategies. Also, such a travel provider may also be lacking in capabilities and needs to take the initiative to join the PAK, relying on the companies that have core and channel resources. These two types of companies are both active participants in the network.

The company's active relationship (AR) is calculated, as shown in Eq. (3).

(3) $${\left[AR_{ii}\right]}_{m}=A^{'}\times A,\;\text{when}r_{ii}=\overset{m}{\underset{k=1}{\sum }}\alpha_{ik}\alpha_{kj}$$

where$\;AR_{ii}$ stands for the diagonal of the adjacent matrix, representing the self-related redundancy, i.e. the total counts of participation in PAK. The more often a company participates in PAK, the more active it will be. That is why $AR_{ii}$ is defined to be the active relationship (AR) of the enterprises.

Complementary relationship

The sum of the column matrix of the adjacent matrix represents how many times a company participates in the PAK. The sum deducted by the number of self-related redundancy represents how many times this company and others participate in the PAK together. To rule out the impact produced by the corporate size, the total number of joint participation in the PAK is divided by the self-related redundancy to obtain other-related redundancy. As the other-related redundancy increases, the companies need more complementary resources. They need to combine many complementary resources in the market to make the business model operate effectively and achieve expected benefits.

The calculations for complementary relationship (CR) are shown in Eq. (4) and in Eq. (5).

(4) $${\left[CR_{ij}\right]}_{m}=A^{\text{'}}\times A,\text{ when }r_{ij}=\overset{m}{\underset{k=1}{\sum }}\alpha_{ik}\alpha_{kj}\;i=1,2,\ldots ,m\;j=1,2,\ldots ,n\;i\neq j$$

(5) $$CR_{ii}=\frac{\sum_{j=1}^{n}CR_{ij}}{AR_{ii}}\;\;i=1,2,\ldots ,m\;j=1,2,\ldots ,n\;\;i\neq j$$

where$\;CR_{ij}$ is the sum of the elements of the row matrix after the multiplication, representing the total counts of the participation in PAK of the company with others together. The higher the count, the more partners the PAK that the company participates in includes. Therefore, that a company has many partners means it has a complementary relationship.

To avoid the impact of the total number of participation in PAK, $CR_{ij}$ is divided by $AR_{ii}$. In this way, $CR_{ii}$ is obtained. The higher the $CR_{ii}$ is, the deeper the complementary relationship is. Meanwhile, other complementary resources are also needed. $CR_{ii}\;$is defined as the complementary relationship (CR).

Step 2 Identify the positions and roles of travel providers

The aforesaid two indicators are used to construct the A (AR)-C(CR) chart with the AR as the x-axis and the CR as the y-axis. The two indexes of each company are calculated, and marked on the A (AR)-C(CR) chart. Further, the corresponding network position in the tourism industry is identified according to the values of the two indicators. The role analysis is conducted based on various characteristics of each position and the positions are properly labeled according to the characteristics.

## 4 Research results

AR and CR are adapted as evaluation indicators. The two indicators are applied to the position analysis in the tourism industry. In total, the companies are divided into four positions. Further, the homogeneity of each position is discussed as role analysis. Lastly, the case from each position is selected to discuss and to verify the feasibility of the evaluation indicators constructed in this study.

### 4.1 Selecting important companies

Firstly, the degree of centrality of 332 companies is calculated through NetMiner software. Based on the degree of centrality, the companies whose relationship is not important are removed. The screening value is 0.2. In this way, 52 important companies are left as analysis data.

### 4.2 Position and role analysis

In order to determine the position of travel providers in the tourism industry, this study calculates first the AR and CR values of the 52 important companies as evaluation indicators, as shown in Table 2. The 52 companies are distributed on the coordinate chart with the AR as the x-axis and the CR as the y-axis. Lastly, the meaning and characteristics represented by each position in the coordinate chart are discussed with individual cases.

**Table 2:** The AR and CR for the travel provider.

Travel providers	AR	CR	Travel providers	AR	CR
South East Tour	78	5.91	China-Times Tour	17	8.00
Lion Tour	74	6.65	Nanlung Tour	17	8.06
Comfort Tour	52	6.42	Fan-Chia Tour	16	8.06
Life Travel	50	6.76	Tunghui Tour	16	5.00
Hsi-Hung Tour	47	7.72	Cts-Travel	16	5.19
Richmond International Travel and Tour	46	7.22	Pudong Tour	15	6.47
Phoenix Tour	45	6.64	Xiamen Air Tour	14	8.64
Gloria Tour	41	6.93	Sunshine Travel	14	7.29
Best Way Tour	38	7.66	Joan Travel	13	8.62
Southwest Travel	38	7.50	Ettours Tour	13	6.69
Pro Tour	36	6.39	Tourone Travel	13	6.38
Peace Tour	35	6.86	Four-King Travel	12	4.75
Ez Travel	34	6.38	Blissful Tour	12	6.92
Perfect Travel	34	7.50	Ch’ Ao Travel	12	6.42
Friendship Tour	31	8.13	Da-Do-Va Tour	11	4.45
Star Travel	31	7.55	Quality Champion Tour	11	5.45
Ever Fun Tour	20	5.52	Jptravel Tour	9	5.22
Cosmo Express Tour	26	6.00	Chang Teng Tour	9	6.22
Golden Team Tour	24	8.04	See-Mark Tour	9	4.67
China-Overseas Travel	21	8.43	Huanyu-Group Travel	9	8.89
Worldwide Travel	21	6.33	Daiei Tour	8	8.50
Artisan Travel	20	7.80	Taiwan Express Tour	8	5.50
Great Voyage Travel	20	8.30	World Tour	8	8.38
Dragon Tour	19	8.11	Ming-Sheng Tour	8	6.25
Bai-Fu Tour	19	8.16	Fu Yang Tour	7	6.43
Ezfly Travel	19	6.74	Tour Travel	5	13.20

#### 4.2.1 Indicator analysis

##### 4.2.1.1 1X-axis: active relationship (AR)

First, the AR is set as the x-axis. If the AR of a travel provider is greater than the average value, its corresponding position on the abscissa is on the right, indicating that the travel provider participates in the PAK more often. This type of travel providers hopes to create a wide range of operating profits, establish a long-term and stable sales network, enhance its operating reputation for individual regional markets, and thus become elite in the eyes of airlines. In this way, multi-faceted support from airlines is gained, such as airlines’ advertising and promoting travel agencies. Thus, the brand awareness of travel providers is enhanced to increase their regional competitiveness, such as Lion Tour and South East Tour.

On the contrary, if the AR value is less than the average, its position on the abscissa is on the left. The corresponding travel provider is not that active in the industry. This type of travel agencies often has a unique regional market, such as Tour Travel and Fu Yang Tour.

##### 4.2.1.2 Y-axis: complementary relationship (CR)

The above-average CR values represent the corresponding travel providers that need a higher degree of complementarities with other companies. This type of travel providers is not able to arrange a tour group alone. However, this type of travel providers, more often than not, has unique or valuable resources that attract others to cooperate with them. Apart from the resources of uniqueness and value, they are lacking in other resources. That is why there is a low possibility that they can organize a tour group alone. Therefore, these kinds of travel providers complement each other by jointly using their own core resources. This type of travel providers is highly complementary to other peer companies, such as Tour Travel and Huanyu-Group Travel.

On the contrary, the below-average CR value means that the corresponding travel provider shares a low degree of complementary relationship with others. This type of enterprises is divided into three categories. The first is the travel provider in its early stage of operation and has not yet expanded its market. As a result, it cannot attract others to cooperate with them successfully. This is also known as the stage on which it has not yet developed enough to participate in the PAK. On this phase, this type of travel providers has a low degree of resource complementarities between others. The second is that the travel provider itself is uniquely positioned in the market; its travel route is extremely unique. Due to the uniqueness, there is only a minority of travel providers who have the same unique positioning in the market. As a result, they meet no suitable partner that can jointly develop unique travel products. This type of travel providers may also be a subsidiary of the group. It does not need to create new resources or increase its market share through the PAK. They may have inexhaustible resources from their parent group. The third type of travel providers has huge financial resources. These companies have a very high level of ability to create their own travel products and organize tour groups themselves. Therefore, they have little need for the cooperation in the form of the PAK.

**Figure 4:**
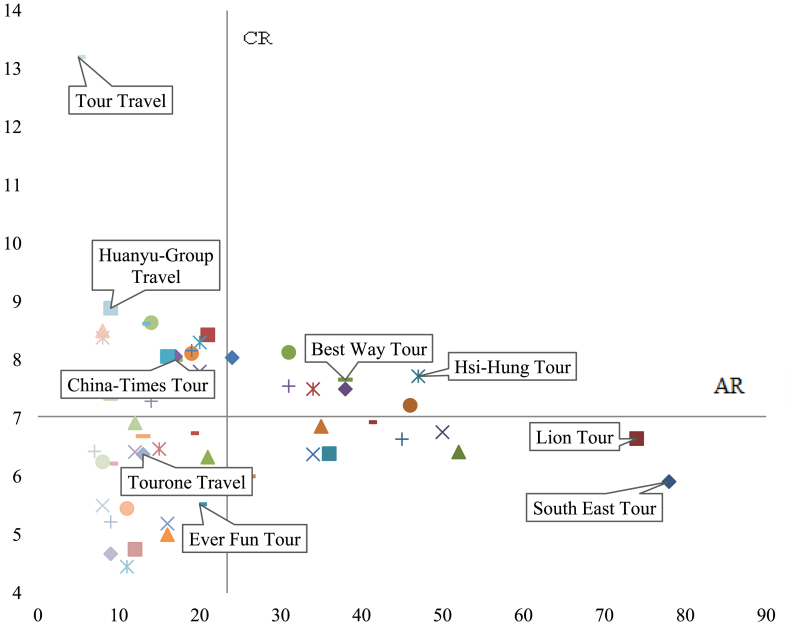
The distribution of the 52 travel providers in the four quadrants on the AR-CR chart.

**Figure 5:**
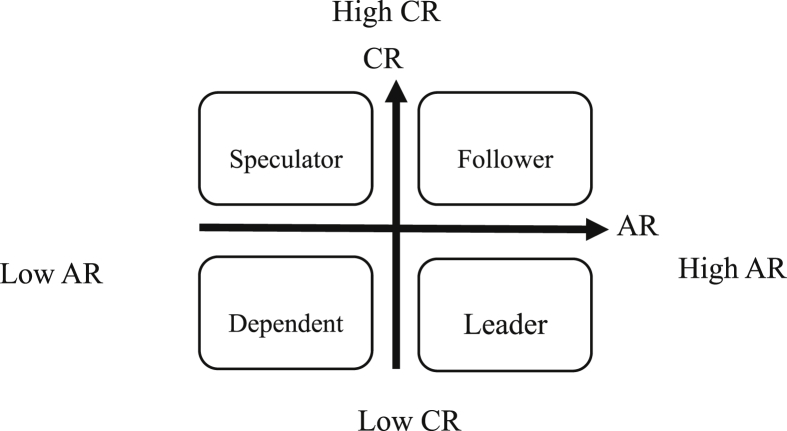
Schematic diagram for the role analysis of AR-CR.

Integrating the aforesaid AR and CR assessment indicators, this study will map the distribution of 52 travel companies on a scatter diagram, as shown in Figure 4, based on their corresponding AR and CR as shown in Table 3 (see Figure 5).

**Table 3:** The positions of the 52 travel agencies.

Quadrant	The travel providers	Counts	Percentage
The first quadrant	Hsi-Hung Tour, Richmond International Travel and Tour, Best Way Tour, Southwest Travel, Perfect Travel, Friendship Tour, Star Travel, Golden Team Tour	8	15.38%
The second quadrant	China-Overseas Travel, Artisan Travel, Great Voyage Travel, Dragon Tour, Bai-Fu Tour, China-Times Tour, Nanlung Tour, Fan-Chia Tour, Xiamen Air Tour, Sunshine Travel, Joan Travel, Chang Teng Tour, Huanyu-Group Travel, Daiei Tour, World Tour, Tour Travel	16	30.77%
The third quadrant	Worldwide Travel, Ezfly Travel, Tunghui Tour, Cts-Travel, Pudong Tour, Ettours Tour, Tourone Travel, Four-King Travel, Blissful Tour, Ch’ Ao Travel, Da-Do-Va Tour, Quality Champion Tour, Jptravel Tour, See-Mark Tour, Taiwan Express Tour, Ming-Sheng Tour, Fu Yang Tour	17	32.69%
The fourth quadrant	South East Tour, Lion Tour, Comfort Tour, Life Travel, Phoenix Tour, Gloria Tour, Pro Tour, Peace Tour, Ez Travel, Ever Fun Tour, Cosmo Express Tour	11	21.15%

### 4.3 Position and role

#### 4.3.1 The first quadrant: followers

The companies at the first quadrant have high values of AR and CR. They have a competitive advantage in the market. Therefore, their performance in the tourism industry is better than their peers, whether in profit, the market share, or in their corporate value. The main reason for this lies in its strategic analysis of their competitive advantages and their adaptation of the PAK as their major tactics to create complementary resources with others and pool complementary resources to jointly create distinctive tourism products. These companies have a dominant role in the market and follow the leading companies. That is why they are named as followers in this study. Among the52 providers, Hsi-Hung Tour, Richmond International Travel & Tour, Best Way Tour, Southwest Travel, Perfect Travel, Friendship Tour, Star Travel and Golden Team Tour fall into this category.

Hsi-Hung Tour established “Tourism Department of Corporate Services and Incentives”. Through this department, the resources of connections are greatly expanded. Further, “Beijing Liaison Office” and “Japan Branch” have been established overseas in order to directly grasp market information and respond to market development needs. The aforesaid information reveals that the business of the company has accumulated some connections and the company hopes to expand the complementary resources for its use. In this way, it will head towards internationalization regarding the operation.

Best Way Tour was established in 1999 to organize tour groups that went to and fro between Taiwan and Hong Kong. Along with the gradually increasing tour groups, it was favored by airlines like China Airlines and became its designated travel agency. With the strong support of China Airlines, Best Way Tour actively expanded its product range to Malaysia, Singapore, Indonesia and Thailand. After it has got a firm foothold, the Republic of Korea and the Mainland China were also included in 2003. The successful business model of the Best Way Tour is favored, for which it is also eligible for the marketing operation center of “Princess Cruises” in Taiwan. It is able to obtain the information of Princess Cruises in time, such as seats, cruise cabins, or cruise information. Even if it has become an upstream company in the industry with core resources, it still needs the sales force of its peers to help reach the goal set for their performance. Therefore, the Best Way Tour is still actively looking for a balance between its partners and its own resources.

#### 4.3.2 The second quadrant: speculators

In the tourism industry, the resources are of a high degree of complementarities. For those enterprises with a low value of AR, they often choose a certain route as their flagship product. In order to consolidate its position in the tourism market, expanding market share is the most important strategy for them, rather than participating in certain PAK to form a strong cooperative relationship with others. These companies play a defensive role in the tourism market. That is why they are called as speculators in this study.

Among the 52 travel providers, the following travel agencies play the role of speculators in this study, namely China-Overseas Travel, Artisan Travel, Great Voyage Travel, Dragon Tour, Bai-Fu Tour, China-Times Tour, Nanlung Tour, Fan-Chia Tour, Xiamen Air Tour, Sunshine Travel, Joan Travel, Chang Teng Tour, Huanyu-Group Travel, Daiei Tour, World Tour and Tour Travel. These companies are highly sensitive to the market. They rely on the analysis of market feasibility as their major strategy and choose a single route to seek deeper development and expansion. Often, they are leaders in the single routes that they focus on. This type of companies often needs to select partners carefully. For example, Mainland China is the second largest country in Worldwide Travel because of its big population. Therefore, long-term cooperation with Mainland China enables them to achieve the same amount of performance even if it depends on the single route involved the Mainland. Usually, these companies pay more attention to connections. Therefore, that necessitates a large amount of energies invested to cultivate and maintain cooperative relationships with others to maintain a stable performance.

#### 4.3.3 The third quadrant: dependents

The enterprises in this quadrant are low in AR and CR values. They are divided into two categories. The first are often dispersed in a diversified market but do not possess key resources themselves. They depend on the key resources given by the upper reaches of the travel industry, such as being agents for airlines or the foreign travel industry in Taiwan, which are differentiated from the mass market. The second are often the subsidiaries of groups, like subsidiaries of parent companies and sibling companies of cross-shareholdings. The groups integrate its own resources and adjust business strategies of each business. In this way, management costs can be significantly reduced and the competitive advantage can be successfully increased. These companies rely on the key resources of upstream manufacturers or their parent companies, so this category is named as dependents.

The first category has diversified market resources. They do not need to develop new market resources through the PAK. The company itself has diversified market resources, and suffices to make profits by organizing tour groups itself. It has the resources that are slightly different from those of the mass tourism market. They often seek the partners with considerable market resources. Therefore, there is a very high degree of scope limitations on the relationship and the direction of cooperation for them, including Ezfly Travel, Da-Do-Va Tour, See-Mark Tour, Worldwide Travel, Pudong Tour, Ettours Tour, Four-King Travel, Blissful Tour, Ch’Ao Travel, Quality Champion Tour, JP travel Tour, Taiwan Express Tour, Tunghui Tour, Ming-Sheng Tour and Fu Yang Tour.

Ezfly Travel is the first large-scale online travel agency in Taiwan. Its main business covers the sales of various travel products, such as airline tickets, room reservations, visas, group tours and various theme tours. Its market uniqueness lies in its integration of overseas global distribution booking system and Expedia Inc. (NASDAQ: EXPE), the world's largest travel website that provides booking options of more than 70 countries and 155,000 hotels. In addition, it has integrated with Japan's company TimeDesign.

The second category often involves the companies with “one-stop” integration capability. Those companies are often subsidiaries of the groups that are developing diversified business. Therefore, their resources often come from the internal supply of the group. Most of their business comes from other subsidiaries of the Group, enabling the customers to enjoy the group's consistently caring services provided by Worldwide Travel. Therefore, they are very likely to control the quality of their services and products. For example, Ever Fun Tour belongs to the Evergreen Group. It enjoys unconditional support from Evergreen regarding advertising, promotion, and circulation, enhancing Ever Fun Tour's brand awareness and increasing its competitive advantage. Tourone Travel and Cts-Travel are also a case in point.

#### 4.3.4 The fourth quadrant: leaders

In addition to stable growth of revenue and profit, these travel providers also actively expand externally, integrate internally, and create competitive capabilities for diversified services. However, such companies have numerous industrial suppliers and seek to promote corporate globalization. These companies have diversified competitiveness at the same time, and play a dominant role in the tourism market. The followers in the industry are competing to imitate them. In this study, these types of companies are called as leaders. Among the 52 companies, the following companies are the leaders, namely South East Tour, Lion Tour, Comfort Tour, Life Travel, Phoenix Tour, Gloria Tour, Pro Tour, Peace Tour, Ez Travel, Ever Fun Tour and Cosmo Express Tour.

Lion Tour is the best travel agency, followed by South East Tour. The director of the Lion Tour Group Wen-Chieh Wang once stated that although Lion Tour Group was not listed as the first travel agency, they have repeatedly set a record in the industry. They are the first to create a 24-hour physical storefront and the first to introduce the program for operations trainees. Through continuous innovation and integration of various market resources, the Lion Tour Group meets the needs of different consumer groups, and thus attracts a large number of clients. After a long period of accumulation and deep cultivation, the Lion Tour Group has established itself as a leader in the tourism industry. Lion Tour also has more than a dozen affiliated companies, some of which focus on international travel services, and so forth. They have diversified channels and have a relatively large group. This has led the other peer companies to show interest in cooperating with it to make up for their insufficient resources.

## 5 Conclusions and suggestions

While traveling abroad, the travel agency is located in a central position of the structure and provides intermediary services between sightseeing suppliers and consumers. The tourism industry is low in barriers to entry and easy to imitate. They need to protect the benefits of their companies through complementary resources. Therefore, travel providers must know clearly the resources that they and their competitors have in the market, which serve as an important reference for decision makers to develop a competitive relationship.

This paper applies the social network analysis to the travel industry. Based on the relationship redundancy, two new indicators, namely, the active relationship (AR) and the complementary relationship (CR) are developed. The travel agencies with similar structures are categorized and the four quadrants created based on AR and CR are clearly defined according to the characteristics of the AR and CR. The construction of these two indicators in this study not only effectively simplifies the tourism industry in which the relationships of travel agencies are relatively complicated, but also clearly shows how the relationships between travel agencies work. In this study, the role of travel agencies is classified as followers, speculators, dependents, and leaders. Travel providers are able to spot their position in the competition in the industry with this research method. Also, they can develop a strategy for future development through role analysis. In this way, they will know themselves and their competitors well, which helps them to survive the fierce competition in the industry.

### 5.1 Suggestions

This study provides the following advice for each of the roles, such as co-opetitive strategies, strategic alliances, and mergers and acquisitions (M&A) strategies.

#### 5.1.1 Strategic alliance

The travel agencies in the first two quadrants are advised to cooperate by forming strategic alliances. In this way, the professionalism of their tourism products can be deepened through the defensive capabilities of speculators. Further, the external core competencies, such as market resources and connections, can be strengthened through the advantages of followers.

**Figure 6:**
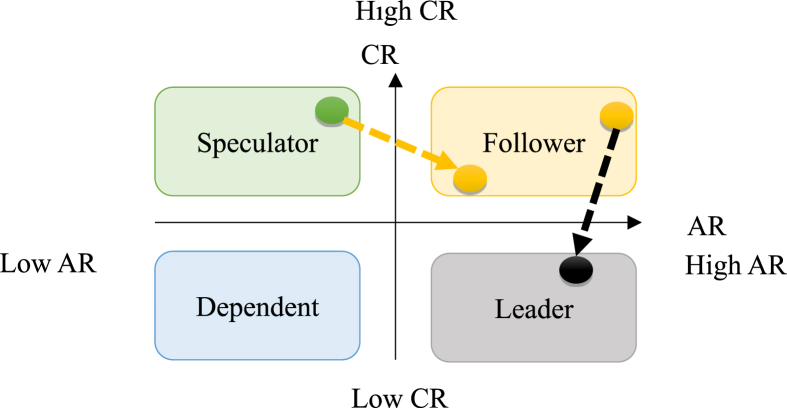
Schematic diagram for strategic alliances.

Followers have a certain degree of core resources in the market; the diversity of their resources is only second to the leaders. If the professionalism in a certain route market of the speculators can be deepened, and the core resources of the followers are to be combined with the professionalism by participating in the PAK jointly. After cultivating their markets, they can migrate to other neighboring countries to develop their markets through the channels owned by the followers that have advantageous resources. In this way, they may direct their attention to the development of an international tourism brand. In this way, not only can they jointly promote a new wave of tourism, but also the followers can rise to the competitive position of leaders, and speculators can rise to followers. This strategy is a way to expand its market and even expand the business to neighboring countries, as shown in Figure 6.

#### 5.1.2 M&A strategy

The entire tourism market in Taiwan seen from a macro perspective, the use of M&A strategies can not only accelerate the acquisition or consolidation of various core resources, but also exert a control over the market so that competitors cannot pose a threat, and thus maintain and protect their own competitive advantages.

**Figure 7:**
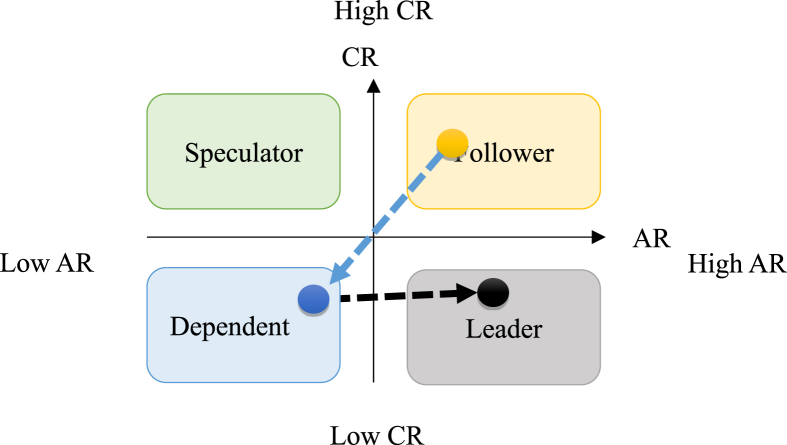
Schematic diagram for M&A strategy.

Through this analysis, the study finds that most dependents have diversified market resources. They also possess sufficient resources to organize tour groups by themselves. Moreover, they are capable of keeping their competitive position. Based on these characteristics, this study suggests that dependents incorporate followers that have advantageous core resources into the group through mergers and acquisitions. This will increase the market share, integrate fully upstream and downstream resources to achieve benefits, and create the largest business value of the enterprise group, heading towards world leadership. As shown in Figure 7, Group A merges the travel agency B into its subsidiary and B becomes the travel agency C under Group A.

### 5.2 Future research

Through the social network analysis, collected data can be used to evaluate the competitive intensity among the groups and internal organizations of major industries. The travel industry is an industry that integrates upstream and downstream businesses. It is in the central position among the sightseeing business. This study only explores the travel agencies and does not involve the PAK between upstream and downstream businesses of the travel industry. Therefore, it is suggested that the future studies be extended in this direction so that travel providers will have practical data and theoretical basis for strategic consideration. That serves as the foundation of corporate transformation, such as the alliance between airlines and the travel agencies, and the analysis of the internal and external resources owned by the travel industry company, exploring the demand for complementary resources and planning future strategic developments.

It is also possible to apply CR and AR to other industries through the basic operation mode of the two indicators. The social network analysis theory can be used to explore the relationships among the actors. After that, the entire industry is viewed based on the meaning of the indicators. Then, past cases are used to verify whether the indicators produce the same effect on other industries.

## Declarations

### Author contribution statement

Yu-hsin Chang: Conceived and designed the experiments; Performed the experiments; Analyzed and interpreted the data; Contributed reagents, materials, analysis tools or data; Wrote the paper.

### Funding statement

This research did not receive any specific grant from funding agencies in the public, commercial, or not-for-profit sectors.

### Competing interest statement

The authors declare no conflict of interest.

### Additional information

No additional information is available for this paper.
